# Stimulus discriminability may bias value-based probabilistic learning

**DOI:** 10.1371/journal.pone.0176205

**Published:** 2017-05-08

**Authors:** Iris Schutte, Heleen A. Slagter, Anne G. E. Collins, Michael J. Frank, J. Leon Kenemans

**Affiliations:** 1 Department of Experimental Psychology and Psychopharmacology, Helmholtz Institute, Utrecht University, Utrecht, The Netherlands; 2 Department of Psychology and ABC, University of Amsterdam, Amsterdam, The Netherlands; 3 Department of Psychology, Helen Wills Neuroscience Institute, University of California, Berkeley, Berkeley, CA, United States of America; 4 Department of Cognitive, Linguistic & Psychological Sciences, Brown Institute for Brain Science, Brown University, Providence, RI, States of America; Universiteit Gent, BELGIUM

## Abstract

Reinforcement learning tasks are often used to assess participants’ tendency to learn more from the positive or more from the negative consequences of one’s action. However, this assessment often requires comparison in learning performance across different task conditions, which may differ in the relative salience or discriminability of the stimuli associated with more and less rewarding outcomes, respectively. To address this issue, in a first set of studies, participants were subjected to two versions of a common probabilistic learning task. The two versions differed with respect to the stimulus (Hiragana) characters associated with reward probability. The assignment of character to reward probability was fixed within version but reversed between versions. We found that performance was highly influenced by task version, which could be explained by the relative perceptual discriminability of characters assigned to high or low reward probabilities, as assessed by a separate discrimination experiment. Participants were more reliable in selecting rewarding characters that were more discriminable, leading to differences in learning curves and their sensitivity to reward probability. This difference in experienced reinforcement history was accompanied by performance biases in a test phase assessing ability to learn from positive vs. negative outcomes. In a subsequent large-scale web-based experiment, this impact of task version on learning and test measures was replicated and extended. Collectively, these findings imply a key role for perceptual factors in guiding reward learning and underscore the need to control stimulus discriminability when making inferences about individual differences in reinforcement learning.

## Introduction

Reinforcement learning refers to the ability of humans and other animals to learn from the outcome of their actions. Actions that lead to a positive outcome are likely to occur more frequently in the future than actions that yield punishment [1]. Motivated or reward-driven behavior is thought to reflect the balance between contributions of different brain systems involved in reward processing and punishment processing, respectively [2]. There is great individual variation in reward sensitivity as well as in sensitivity to signals of punishment. In other words, humans differ in motivational style.

Reinforcement learning tasks are used to assess motivational style often by testing individual differences in reinforcement learning overall [3] or whether one is better at choosing stimuli that likely lead to reward or better at avoiding stimuli that likely lead to punishment (e.g. [4–8]). The probabilistic selection task (PST; [5]) has been used many times to assess effects of patient populations and dopamine medications on learning from positive vs. negative feedback, and individual differences in these measures have been repeatedly related to neural and genetic measures associated with striatal and dopamine function (e.g. [5,9–19]).

In the PST, individuals first learn to choose which stimulus characters of three different stimulus pairs are most likely to be rewarded. Stimulus A is associated with positive feedback on 80% of AB pair trials whereas stimulus B is associated with negative feedback on 80% of AB trials. Outcome contingencies within the other pairs (CD and EF) are less consistent. Choosing C and D is followed by positive and negative feedback, respectively, in 70% of the trials. E and F are paired with positive and negative outcomes, respectively, in 60% of the trials. During the test phase all possible combinations of training stimuli are presented in pairs. Subjects who more often avoid stimulus B in novel test pairs during the test phase, relative to choosing A, are classified as negative learners. In contrast, subjects who more often choose stimulus A in novel test pairs during the test phase, relative to avoiding B, are classified as positive learners. These learning biases have been related to individual differences in event-related potential (ERP) and neuroimaging components of feedback processing, dopaminergic medication status and genetic polymorphisms associated with dopamine function (see references above). These observations are in line with the notion that performance on the PST and other RL tasks captures effects of manipulations (e.g. drugs) and individual differences in motivational style related to the reward valuation system itself.

However, thus far the modern reinforcement learning literature has not investigated whether overall learning–and especially positive vs. negative learning–may depend on perceptual aspects of the stimuli themselves. An important aspect is discriminability, defined here as the extent to which a stimulus stands out from the background and from other stimuli due to its distinctive global and/or local features (e.g. shape or color). For example, stimuli that are perceptually more discriminable than others may be easier for participants to remember to select once they have been rewarded. Salience or discriminability may also affect preference (ratings of trustworthiness of faces; [20]). Furthermore, it has been shown that discriminability and preference interact to affect free choice in a classification task [21]. In addition, there is a collection of older literature describing how learning of the affective value of a stimulus is modulated by its salience. This holds in particular for salience as conveyed by the relative novelty or unfamiliarity of the stimulus. The relation between salience and the extent to which a stimulus affords learning has become manifest in classic phenomena such as blocking (e.g., [22]) and latent inhibition [23]. It is reflected in incidental learning about salient relative to non-salient items as described already in 1933 by von Restorff ([24]; see also [25]), and in relatively strong physiological orienting reactions to novel stimuli predicting subsequent affective learning about these stimuli [26,27].

Also recent research indicates that our choice between alternatives is not only affected by reward value but also by stimulus salience, and that perceptual and reward properties may influence decision making via common neural mechanisms [28–32]. Indeed, Cavanagh et al. [33] have shown in the test phase of the PST that eye gaze time toward a stimulus increases the chances of selecting it, regardless of its value. This body of research mainly pertains to conditions in which reward contingencies have already been learned but salience might also influence learning about reward itself. The latter is not only of profound theoretical significance by itself but also a potential concern as assessment of motivational style often requires comparison of learning bias across different task conditions, which may differ in the relative salience or discriminability of the stimuli associated with more and less rewarding outcomes, respectively.

**Fig 1:**
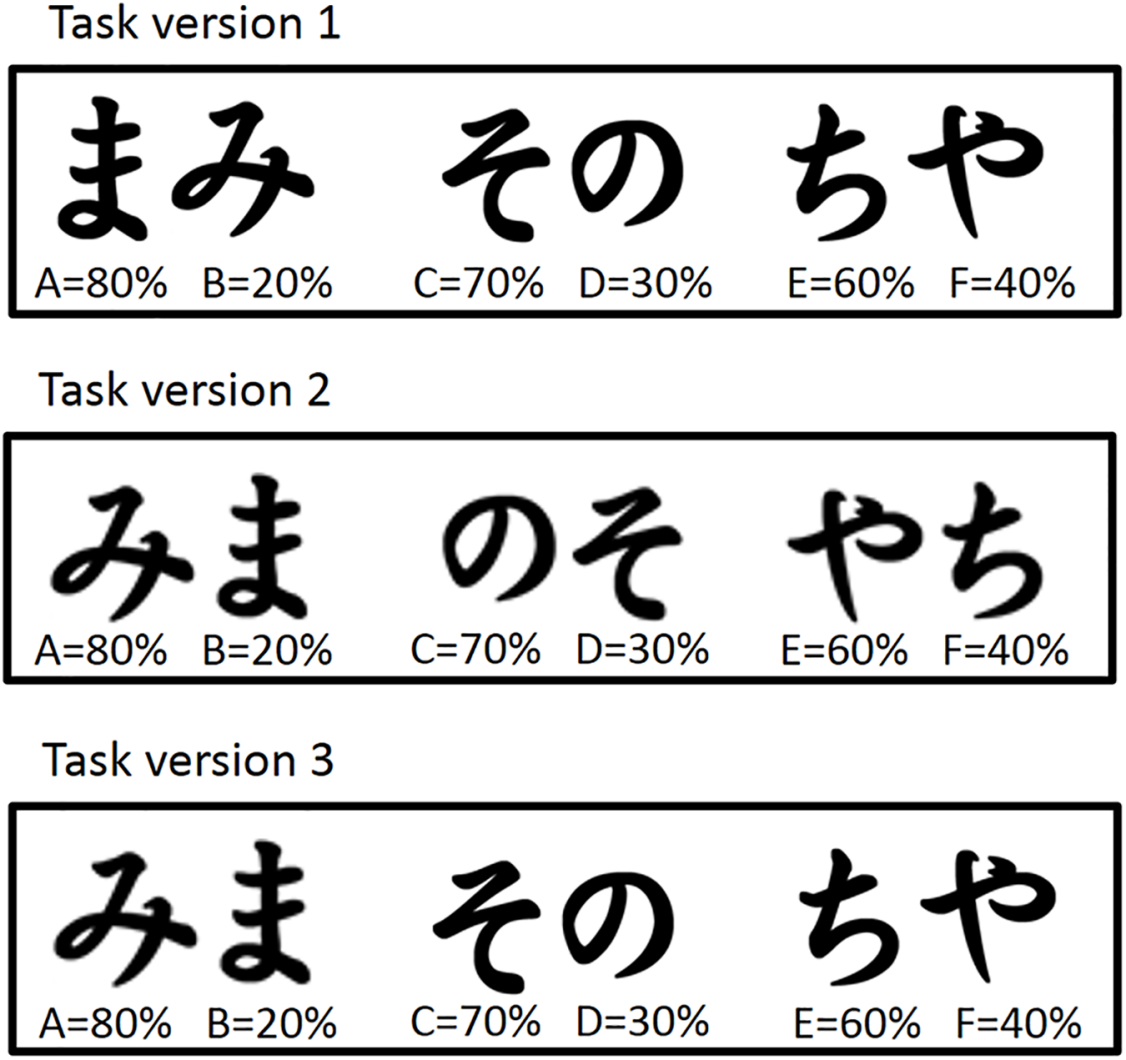
Hiragana stimuli used in the probabilistic learning task in experiments 1a and 1b and experiment 3. Each pair of stimuli was randomly presented in separate trials. During each trial participants chose one of the stimuli of the pair. Feedback following participant’s choice was determined probabilistically. Reward probability (indicated below each stimulus) differed between characters. Task version 1 and 2 differed with respect to the characters associated with more probable positive and more probable negative feedback, respectively. Specifically, in task version 2, Hiragana stimuli were switched within the AB, CD and EF pair. Half of the participants were subjected to version 1 and the other half to task version 2. In experiment 3, a task version was used for which only the Hiragana stimuli within the AB pair were switched.

The aim of the current study was therefore to investigate if classification as a positive or negative learner in the PST task solely reflects variation in motivational style, or may also reflect variation in the extent to which aspects of the stimulus configuration afford learning from the stimuli in general. To this end, in two experiments (Experiment 1a and 1b), which only differed in the time available to respond, participants performed one of two versions of the PST. These versions differed in assignment of physical characters to the functional A-F categories as discussed above. We used Hiragana letter stimuli (see Fig 1) as typically used in the PST as described by Frank et al. [5,14]. For both task versions we used the same stimuli and a fixed Hiragana- A-F mapping. However, critically, in the second version, compared to the first, Hiragana letters were switched within the AB, CD and EF pairs. Given prior work suggesting that the PST task is sensitive to individual differences in learning bias that reliably relates to genetic and neural measures when randomizing or counterbalancing stimulus assignments, we expected to find no effect of specific fixed assignments on the proportion of positive and negative learners across versions. However, to foreshadow our results, in both experiments, we found that stimulus-to-feedback mapping strongly affected the learning curves of the individual stimuli and subsequent positive/negative classification in the test phase. Moreover, a subsequent experiment showed that some Hiragana stimuli were more discriminable (here referring to the extent to which these stimuli stood out from the environment specifically because of their shape) than others. This indicates that for any arbitrary configuration of stimuli and stimulus-contingency mappings, classification of reinforcement learning does not depend only on individual differences in positive and negative learning mechanisms themselves, but can also be affected by variation in the extent to which stimuli afford learning in general. This issue concerns not only the specific probabilistic learning task addressed presently, but also other varieties typically using a single set of stimuli for each condition such as those showing similar effects of dopaminergic manipulations on learning from reward or punishment [4,7,8]. Furthermore, this issue may be especially problematic when making inferences about positive/negative learning on the individual level. It should, however, be emphasized that the studies cited above using the PST have all randomized and counterbalanced the stimulus-to-feedback mappings and hence these reported findings cannot be attributed to differences in discriminability between the stimuli.

Indeed, a key prerequisite for interpreting test phase choices in the PST is that feedback experiences during the training phase are similar across subjects [5]. That is, assessment of a given subject/group’s ability to generalize positive /negative learning about A/B in the test phase requires that this subject/group had experienced comparable positive and negative associations for these stimuli during learning compared to other subjects/groups. We therefore additionally compared performance during the training phase between both task version groups. We found that task version had strong effects on learning curves, such that subjects were more reliable in selecting rewarding characters that were more discriminable, leading to differences in experienced reward probability for the critical items across versions. Furthermore, to replicate the findings from experiment 1, the effect of stimulus-to-feedback mapping on feedback learning was additionally investigated in a large-scale web-based experiment (experiment 3). Experiment 3 furthermore addressed the possibility that feedback learning primarily depends on the extent to which salient stimuli uniquely stand out relative to non-salient stimuli in conveying either positive or negative feedback.

## Methods–experiments 1a and 1b

### Participants

Thirty-one subjects participated in experiment 1a and 25 new subjects participated in experiment 1b. All subjects were recruited through posters at the campus of Utrecht University and were given a financial compensation of 3 Euro per half an hour or study credits. All subjects declared to have normal or corrected-to-normal vision and all subjects were unaware of the aim of the study. Participants were asked to refrain from caffeine use and smoking on the day of the experiment. Exclusion criteria were a history of psychological or neurological disorders, age below 18 years, and caffeine use or smoking at the day of and before the experiment. This experiment was approved by the local ethics advisory board of the Faculty of Social Sciences of Utrecht University. Written informed consent was obtained and participants were treated according to the Declaration of Helsinki.

Nine participants in experiment 1a were discarded during analysis because they did not satisfy performance criteria during the learning phase (see section task and procedure). The final sample consisted of 22 participants (experiment 1a). Half of them was assigned to version 1 of the probabilistic learning task (mean age: 22.6, SD: 3.0, range: 19.1–27.8 years, 9 females) and the other half to version 2 of the probabilistic learning task (mean age: 23.0, SD: 2.3, range: 19.7–26.6 years, 9 females). The two task version groups were matched for age and gender.

Of the 25 participants in experiment 1b, five were discarded during analysis because they did not satisfy performance criteria during the learning phase and/or test phase (see section task and procedure). The final sample of experiment 1b consisted of 20 participants. Half of them finished version 1 of the probabilistic learning task (mean age: 20.7, SD: 1.3, range: 18.5–23.0, 8 females) and the other half finished version 2 (mean age: 21.8, SD: 2.0, range: 19.2–24.9, 8 females). The two task version groups were matched for age and gender.

### Task and procedure

#### Experiment 1a

Upon arrival at the lab, subjects received information on the procedure of the experiment and written informed consent was obtained. Participants were seated in a chair 85 cm in front of a computer screen. Presentation of instructions and stimuli was controlled by Presentation® software (version 16.0, http://www.neurobs.com). Participants were subjected to a slightly adapted version of the probabilistic learning task as described in the article by [14]. For a subset of participants EEG was recorded during task performance (not discussed in this article). Participants were assigned to one of two task versions (1 and 2) which differed in assignment of Hiragana characters to elements A to F. Fig 1 represents the complete mapping of specific Hiragana characters on A to F for the two task versions. Note that in version 2, relative to version 1, Hiragana-A-F mapping was only switched within pairs (the pairs being AB, CD, and EF). Letter stimuli within a frame of 3° by 3° were white on a black background. One character of each pair was presented on the left side of the screen and the other character on the right side of the screen (center-to-fixation distances were 5 degrees of arc). The left or right character was selected by pressing the “z” or “m” key, respectively, on a qwerty keyboard. The position of each Hiragana stimulus was pseudo-randomized across trials.

The task consisted of two phases, a learning phase and a test phase. During the learning phase, subjects were exposed to a maximum of seven training blocks each consisting of a random sequence of 10 repetitions of each of the six Hiragana combinations (60 trials in total). Each trial consisted of a fixation cross (a white plus sign) presented for a random duration between 250 and 750 ms, a pair of Hiragana stimuli presented for 750 ms, followed by a blank screen for 250 ms, followed by visual feedback presented for 600 ms. On each trial subjects chose one of the stimuli and feedback was presented regarding their choice. If participants did not respond within 1000 ms, the message “no response made” was presented. The feedback stimulus was either a circle to signify that the choice had been ‘correct’ (positive feedback), or a triangle for a choice that had been ‘incorrect. Choosing the A stimulus resulted in positive feedback on 80% of the AB trials, and in negative feedback on 20% of the trials. For choosing the B stimulus these percentages were 20% for positive and 80% for negative feedback. The percentages for positive and negative were 70 and 30 for choosing C, 30 and 70 for choosing D, 60 and 40 for choosing E, and 40 and 60 for choosing F, respectively. Participants were told in advance that feedback would be probabilistic (the literal wording being ‘possibly incorrect’) and that they should select the stimuli that were most likely to be rewarded.

Before advancing to the test phase, subjects needed to meet a performance criterion. During a training block participants needed to choose at least 65% A, 60% C and 50% E stimuli (percentage of the total number of AB, CD and EF trials in a block, respectively). After each training block it was evaluated whether the performance criterion was met. If the performance criterion was not met, the next training block was presented. A minimum of two and a maximum of seven training blocks were presented. Participants who did not meet the performance criterion at the end of the seventh block (this held for 9 participants) were excluded from further participation, to make sure that every subject was at the minimum required performance level at the start of the test phase.

Preceding the training phase, participants were told that in addition to the stimulus pairs from the training phase, new combinations of the same Hiragana characters would be presented. They were instructed to again choose the character that would most likely be rewarded, but that feedback would no longer be given. The test phase consisted of three blocks of 90 trials, resulting in 270 trials. During each block all 30 (6 letters, with 2 possible positions for each letter) stimulus combinations including novel and training pairs were randomly presented three times. Each trial consisted of a fixation cross (same as during the learning phase), the stimulus pair presented for 1000 ms, and a blank screen presented for 950 ms. No feedback was given on participant’s choice. If participants did not respond within 1000 ms after stimulus onset, the message “no response made” was presented. After completion of the test phase (or the seventh training round in case the performance criterion was not met) participants were subjected to an awareness questionnaire to assess whether participants were aware of the reward-punishment contingencies of each character. Finally, subjects were paid and dismissed.

#### Experiment 1b

The task in Experiment 1a was adapted from a task used in an EEG study by [14], in which a response window of 1000 ms was implemented, which is short compared to other studies. To address the possibility that the relatively short response window induces a stronger influence of stimulus-related factors, an additional experiment was run in which the maximum amount of time participants had to respond was changed to 4000 ms. Thus, the task and procedure were identical to the task and procedure of experiment 1a, except that participants had a maximum of 4000 ms after stimulus onset to respond to the Hiragana stimuli in both the training phase and the test phase, as comparable to [5].

### Data reduction and analysis

Participants who did not satisfy the performance criterion at the end of the seventh training round were excluded (9 participants in experiment 1a (5 in version 1 and 4 in version 2) and 2 participants in experiment 1b). Furthermore, participants who did not choose more than fifty percent A on the AB trials of the test phase (AB) were not analyzed further (0 participants in experiment 1a and 3 participants in experiment 1b). The overall percentage (of response trials, excluding no-response and double-response trials) of choosing stimulus A and avoiding stimulus B in novel test pairs (A or B paired with either C, D, E or F) during the test phase was calculated for each subject. Participants were categorized as positive learner if percentage choosing A was higher than percentage avoiding B. Participants were categorized as negative learner if percentage avoiding B was higher than percentage choosing A. A t-test was conducted for each task version group to assess whether participants were more inclined to choose stimulus A or to avoid stimulus B (in other words, a within-subjects test comparing % choose A in novel test pairs to % avoid B in novel test pairs). A Pearson Chi-square test (or Fisher's exact test if appropriate) was conducted to investigate whether there was a relationship between task version (version 1 or version 2) and categorization as positive or negative learner.

To analyze performance during the training phase, for each participant the percentage choices for the most rewarded stimulus (of response trials, excluding no-response and double-response trials) within each of the training pairs was computed. This was done for the first training round and for the last training round (i.e., the round before advancing to the test-phase, which was individually determined). These percentages were entered in a mixed MANOVA with stimulus pair (AB, CD, EF) and round (first, last) as within-subject factors and task version (1, 2) as between-subjects factor.

## Results–experiments 1a and 1b

### Experiment 1a

**Fig 2:**
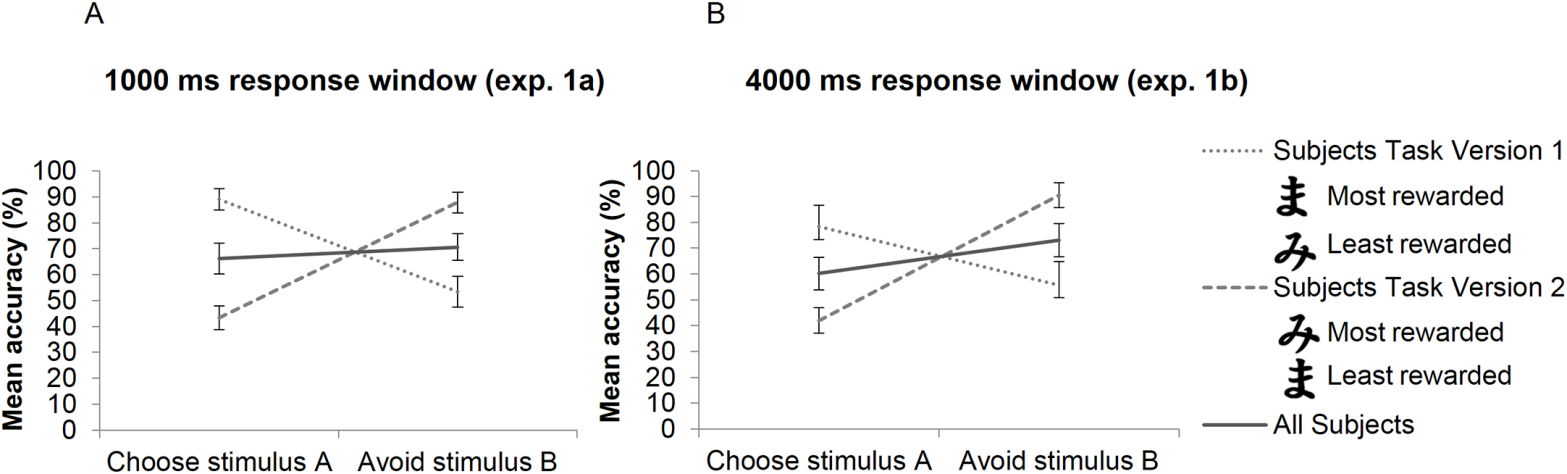
Stimulus properties affect extent of learning from positive vs. negative feedback. This figure shows stimulus A and B accuracy (choosing the most rewarded stimulus) in the test phase for both task versions in experiment 1a (panel A) and experiment 1b (panel B). In both experiments (i.e., regardless of the length of the response window), subjects who completed task version 1 were overall better at choosing A, whereas in version 2 subjects were better at avoiding B. These findings indicate that stimulus salience may strongly bias value-based probabilistic learning. Error bars represent ± 1 SE.

Ten out of 11 participants who finished version 1 of the probabilistic learning task were better at choosing A than avoiding stimulus B in novel test pairs and were consequently classified as positive learner (within-group t-test for the difference between choosing A and avoiding B: t(10) = 4.31, p = .002, d = 1.3). Ten out of 11 participants who finished version 2 of the task were categorized as negative learner because of their greater accuracy in avoiding stimulus B compared to choosing A (within-group t-test: t(10) = -5.39, p < .001, d = -1.62). Mean percentage A and B accuracy of the two task versions is shown in Fig 2A. A Pearson Chi-square test confirmed the relationship between task version and categorization as positive or negative learner (χ^2^ (1) = 14.73, p < .001). The corresponding phi coefficient was 0.82 (p < .001), which represents a high association between task version and categorization as positive or negative learner.

**Fig 3:**
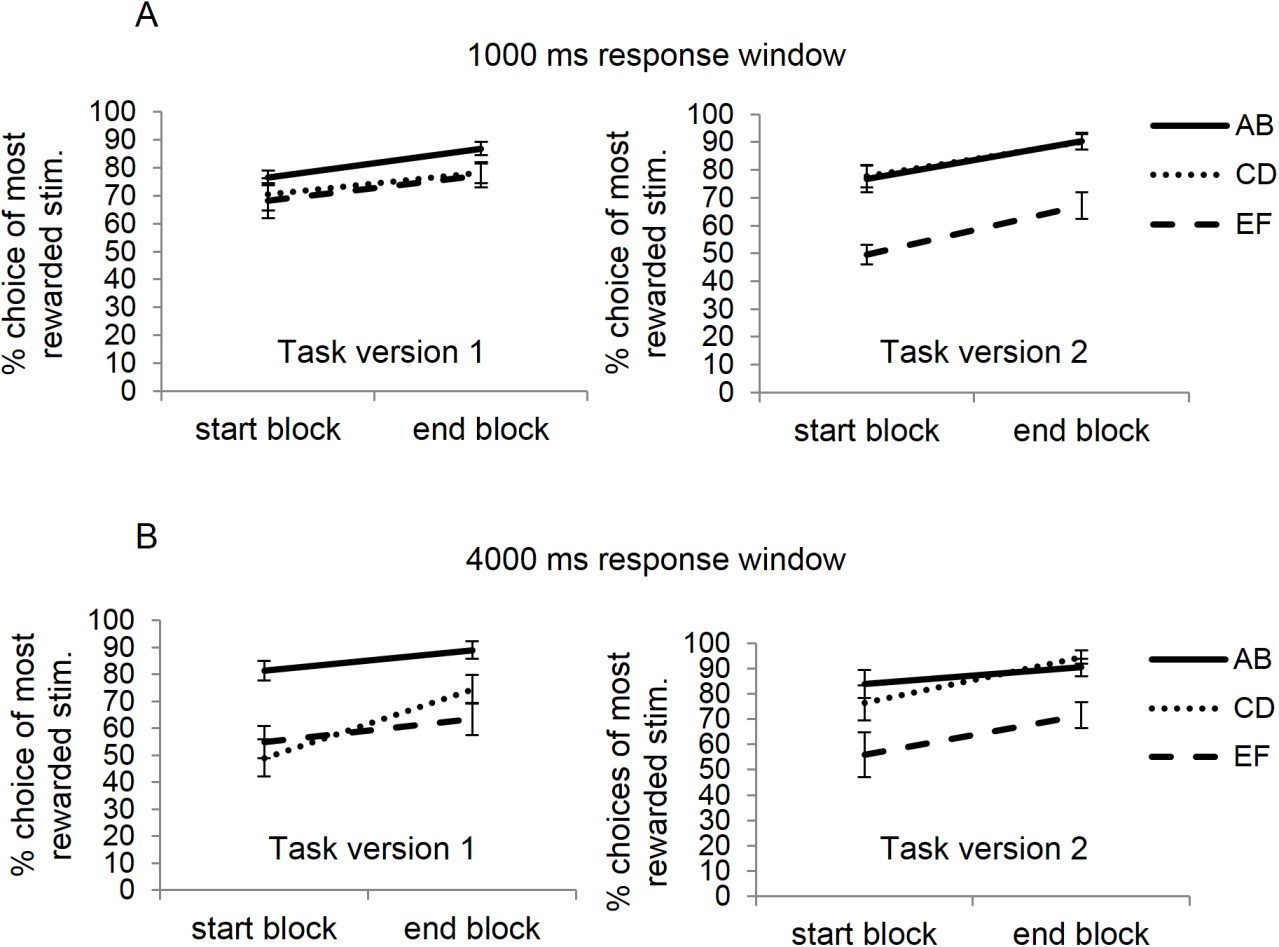
Stimulus properties affect choices during the training phase of the probabilistic learning task. The figure displays accuracy (choosing the most rewarded stimulus) for each of the three Hiragana pairs for both task versions in experiment 1a (panel A) and 1b (panel B). In task version 2, subjects performed better at both the AB and CD pair relative to the EF pair, compared to task version 1. This pattern was consistent across both experiments. Furthermore, subjects in task version 1 of experiment 1b performed better at the AB pair relative to the other pairs, compared to task version 2.

To determine whether these differences were accompanied by differences in experienced reward outcomes during learning (e.g., if better choose-A performance in version 1 was associated with more instances of positive feedback for choosing A compared to other stimuli), we analyzed performance in the training phase. Fig 3A displays training performance for each of the Hiragana pairs for task version 1 and task version 2, respectively. The repeated-measures MANOVA comparing training performance between task versions revealed a main effect of block, indicating that individuals were better at choosing the most rewarded character of each pair during the last compared to the first block of the training phase (F(1,20) = 31.4, p < .001, *η*_p_^2^ = .611). Importantly, there was also an interaction between task version and stimulus pair (F(2,19) = 4.39, p = .027, *η*_p_^2^ = .316). Follow-up MANOVAs for each task version separately showed that there was a significant main effect of pair for task version 1 (F(2,9) = 4.36, p = .047, *η*_p_^2^ = .492) and for version 2 (F(2,9) = 21.93, p < .001, *η*_p_^2^ = .830). Post-hoc tests revealed that in version 1, AB performance was greater than CD performance at near significance (p = .052); whereas no such differences were observed in version 2 (p = .946), although AB and CD performance were both significantly better than EF performance (p’s < .001). These findings suggest that enhanced choose-A vs. avoid-B performance in version 1 might relate to relatively greater experience of positive feedback for A and hence more learning about the positive value of A compared to e.g., stimulus C, whereas better avoid-B performance in version 2 might relate to relatively worse performance in AB and hence more negative feedback for B. The larger sample sizes in experiments 3 (and replication in experiment 1b) below further support this intuition.

### Experiment 1b

Of the ten participants subjected to task version 1, eight participants were classified as positive learner, because of their greater accuracy in choosing stimulus A in novel test pairs, compared to avoiding B. All ten participants subjected to task version 2 were classified as negative learner, because of their greater accuracy in avoiding stimulus B in novel test pairs, compared to choosing A. A t-test conducted for each task version group showed that participants subjected to task version 2 were significantly better at avoiding stimulus B than choosing A in novel test pairs (t(9) = -6.07, p < .001, d = -1.92). Participants subjected to task version 1 tended to be better at choosing stimulus A in novel test pairs (t(9) = 1.52, p = .164, d = 0.48). Mean percentage A and B accuracy are shown in Fig 2B for each task version separately. Results of the Fisher's exact test indicated a strong relationship between task version and categorization as positive or negative learner (p = .001). The corresponding phi coefficient was 0.82 (p = .001).

Results in the training phase were also similar to Experiment 1a. Fig 3B displays training performance for each of the Hiragana pairs for task version 1 and task version 2, respectively. As expected, the repeated-measures MANOVA comparing training performance between task versions indicated overall better performance during the last compared to the first round of the training phase (F(1,18) = 37.24, p < .001, *η*_p_^2^ = .674). The interaction between task version and stimulus pair was again significant (F(2,17) = 4.37, p = .029, *η*_p_^2^ = .339). Follow-up MANOVAs for each task version separately showed a significant main effect of stimulus pair for both versions (task version 1: F(2,8) = 22.58, p = .001, *η*_p_^2^ = .850; task version 2: F(2,8) = 9.23, p = .008, *η*_p_^2^ = .698). Replicating the results of experiment 1a, performance in the AB pair was significantly better compared to both of the other pairs in task version 1 (p’s ≤ .003), whereas in version 2 AB performance did not differ from CD (p = .71) while performance for both the AB and CD pair was enhanced relative to the EF pair (p’s ≤ .004). In version 1 there was no difference in performance between the CD and EF pair (p = .673).

## Discussion–experiments 1a and 1b

The results of experiment 1a show that classification as positive or negative learner was strongly affected by the stimulus-to-feedback mappings. We replicated this finding in experiment 1b in which subjects were given more time to respond. Importantly, this biased classification pattern during the test phase was preceded by differences in performance between the task version groups during training. Participants subjected to version 1 of the task chose the rewarded A over B more reliably than they chose C over D or E over F. In contrast, participants subjected to version 2 consistently chose the more rewarded stimulus C over D equally often as they chose A over B. Because of that, the difference in amount of experienced positive feedback for A relative to C was reduced in version 2 relative to version 1 (in which participants experienced more positive feedback for A than for C). In contrast, the difference in amount of experienced negative feedback for B relative to D was increased in version 2 relative to version 1. These differences in feedback learning or experience may have contributed to the biased classification as positive/negative learner. For example, in version 2, the reduced amount of positive feedback for A relative to C may have resulted in more choices for C over A during the test phase, compared to version 1. Similarly, version 2 enhanced the degree to which subjects experienced more negative feedback for stimulus B than D. Together, these effects would contribute to differential ability to choose A and avoid B across versions. The training results will be more extensively interpreted in the general discussion.

Hence, we hypothesized that in the pair-wise setup of the probabilistic learning task some of the characters may have been more easily discriminable than others. This would contribute to relatively enhanced learning for easily discriminable letters about contingent mainly positive or mainly negative feedback. In this scenario, choices during learning and test phases would not reflect 'positive' or negative' learning styles exclusively, but also the extent to which an easily discriminable stimulus was mainly associated with positive or negative feedback. We therefore explicitly investigated whether some stimuli of the set used in Experiment 1 are more discriminable than others, in a follow-up Experiment 2. Each combination of two stimuli in a random sequence was framed in the context of a standard choice-reaction-time decision-making experiment. We assume that on each trial the evidence for either stimulus identity drifts until a criterion is reached of sufficient evidence to trigger a response. We further assume that the more discriminable the two stimuli are, the more quickly the evidence criterion is reached for either stimulus. Therefore, for pairs consisting of one or two highly discriminable stimuli, reaction times for either of the stimuli will be relatively short, and so will the average reaction time across all trials in that sequence. A systematic difference in discriminability among the six characters would then be revealed by comparing reaction times averaged across all trials within each combination of two characters, subsequently averaged across all combinations containing that specific stimulus.

## Method–experiment 2

### Participants

We included 10 new participants in the second experiment (mean age: 23, range: 19.5–30.9 years, 6 females, 9 right-handed). The sample consisted of trainees and subjects recruited through posters. All participants declared not to have consumed caffeine or nicotine on the day of the experiment. None of the subjects was receiving mental health treatment and none of them was aware of the aim of the experiment. All subjects had normal or corrected-to-normal vision and gave written informed consent. A financial compensation of 3 Euro per half an hour or study credits was given to externally recruited subjects. Participants were treated according to the Declaration of Helsinki. This experiment was approved by the local ethics advisory board of the faculty of social sciences of Utrecht University.

### Task and procedure

Upon arrival at the lab, subjects received information on the procedure of the experiment and informed consent was obtained. As in experiment 1, participants were seated 85 cm in front of a computer screen. Presentation of instructions and stimuli was controlled by Presentation® software (version 16.0, www.neurobs.com). One non-Dutch participant received task instructions in English, the others in Dutch. The same Hiragana characters (white on a black background within a frame of 3° by 3°) were used as in experiment 1.

**Fig 4:**
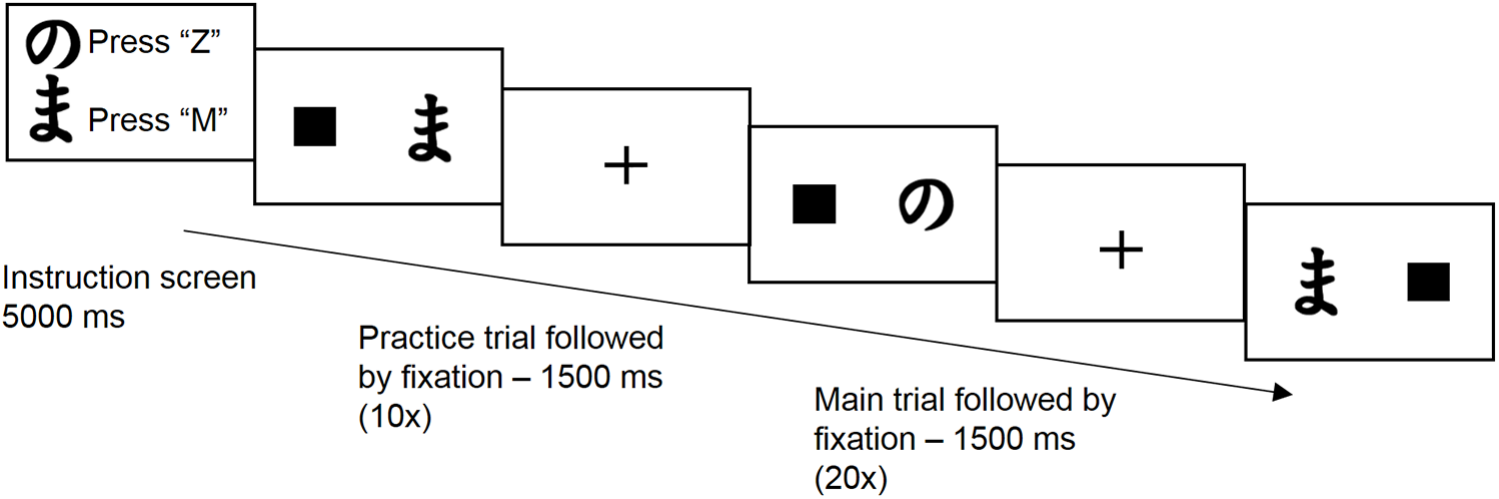
The discrimination task used in experiment 2. The six Hiragana characters were exhaustively combined in separate pairs. Participants were instructed to respond as fast and accurate as possible to each of the stimuli of the current pair according to the instructions presented at the beginning of each block. Note that stimuli and text are presented in black for illustrational purposes, in contrast to the actual task.

An example of the task is shown in Fig 4. Each block of the discrimination task started with a visual instruction of 5 seconds showing which button to press in response to which specific Hiragana stimulus. During each block the “z” key had to be pressed when one of the stimuli of the current pair was presented and the “m” key had to be pressed when the other stimulus was displayed. A random Hiragana character of the current pair could either be presented on the left or right side of the screen and was accompanied by a square on the opposite side. Participants were instructed to respond to the stimuli as fast and accurately as possible. It was emphasized that they had to react to the stimulus character independent of its location on the screen.

Presentation and position of stimuli was counterbalanced across trials. A block started with 10 training trials to practice responses to the Hiragana stimuli of the current block and was followed by 20 experimental trials. A message was presented between the last practice trial and the first experimental trial stating “This was a practice round. The main round will follow now”. The task consisted of 30 blocks presented in random order comprising all combinations of the aforementioned Hiragana characters and corresponding button presses. Subjects pressed the spacebar to continue with the next block. There was opportunity for a small break after the 10th and 20th block. After completion of the task participants were paid (if applicable), thanked for their participation and dismissed.

### Data reduction and analysis

The total number of trials for each subject amounted to 600. Individual trials with RTs > 1000 ms, incorrect responses and multiple responses were removed from the analysis. This pertained to 8.6% of the trials across subjects (range: 3.5–21.2% of the trials per subject). Out of a total of 1200 cells (10 subjects x 30 blocks x 2 stimuli x 2 positions), 17 cells (1.4%) did not contain data due to zero correct and valid responses for those cells (range: 0–5.8% empty cells per subject). Empty cells were replaced by values corresponding to the same block and stimulus (e.g. A versus B block, presentation of stimulus A), but with reversed hand mapping. The number of empty cells did not differ across the 20 conditions (5 combinations x 2 hand mappings x 2 positions). For each subject RTs were averaged (across at maximum 5 trials) for each character within each stimulus combination, for left and right hand mapping and incongruent and congruent (i.e., screen position with respect to response hand) presentations separately. Next, for each subject all reaction times were averaged within each stimulus combination. Data were subsequently averaged across all stimulus combinations containing one specific stimulus (i.e., all combinations containing stimulus A, all combinations containing B, etcetera). Finally, six average RTs for each subject (one for each Hiragana character) were entered into a MANOVA. Post-hoc pairwise comparisons were conducted in order of RT difference between two Hiragana characters, starting with the pair with the largest difference in RT, followed by the pair with the next largest difference, etcetera. This procedure was continued until a non-significant difference was obtained.

The data were also analyzed in a different manner, namely by only including trials with the presentation of a specific stimulus and by subsequently averaging these data across all conditions with that specific stimulus, see S1 File (Alternative analysis).

## Results–experiment 2

**Fig 5:**
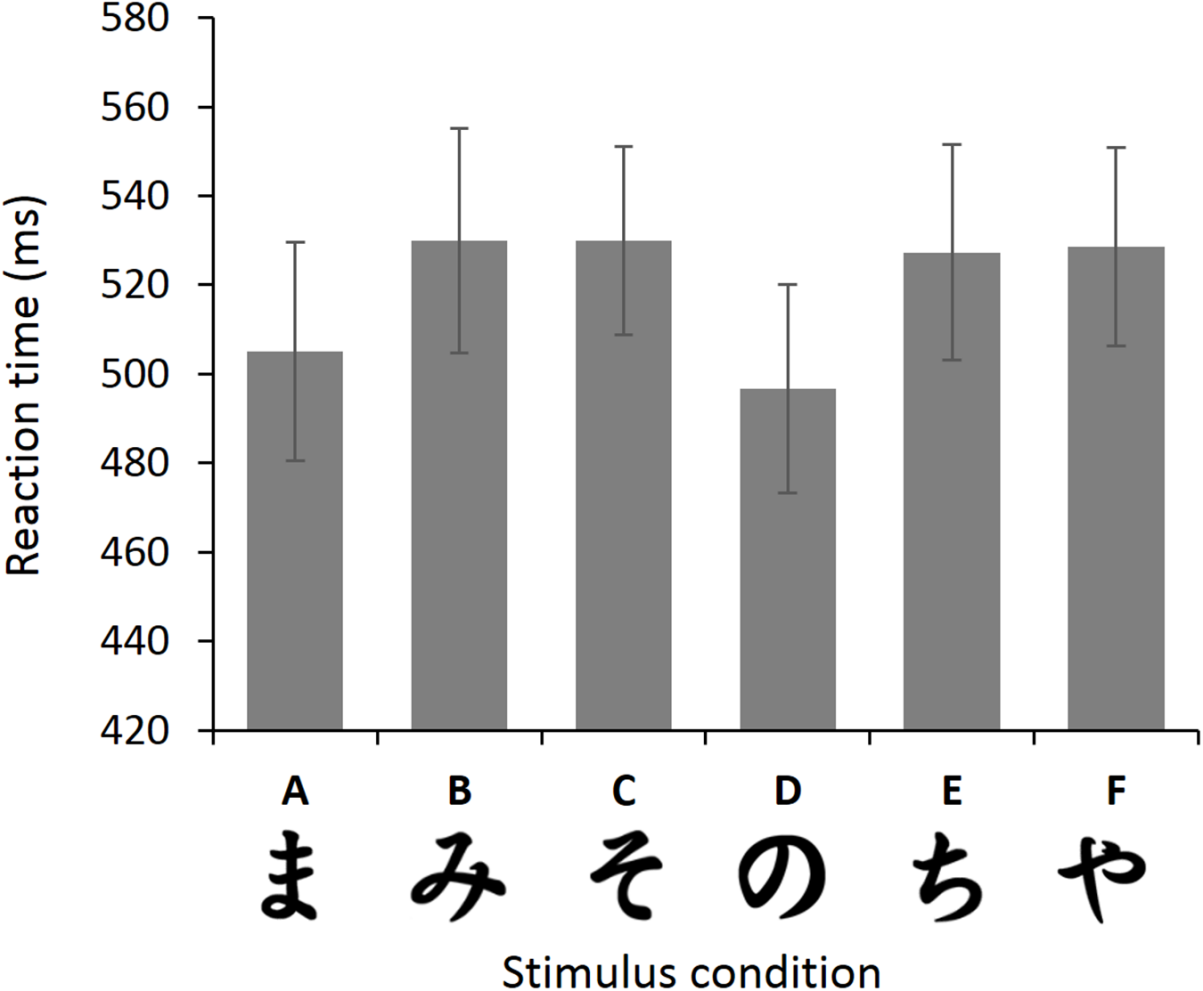
Reaction times to each of the Hiragana stimuli. Subjects reacted significantly quicker to pairs containing stimulus A and D compared to pairs containing the other characters. Error bars represent ± 1 SE. Note that error bars depict variation per condition, not the difference between conditions (as used in the statistical error term).

A repeated-measures MANOVA revealed a significant main effect of stimulus character on RT (F(5,5) = 29.91, p = .001, *η*_p_^2^ = .968). Post-hoc pair-wise comparisons in order of RT difference between two Hiragana characters showed significantly shorter RTs for D compared to B, C, E and F (all p values ≤ .001) and for A compared to B,C,E and F (all p values ≤ .008). Results are shown in Fig 5.

## Discussion–experiment 2

Results of the discrimination experiment clearly show that RTs differ between Hiragana stimuli. Stimulus A, which was the most likely to be rewarded in version 1 of our reinforcement learning experiment and least rewarded in version 2 of that experiment seems more easily discriminable, as evidenced by faster responses for pairs containing this stimulus compared to pairs containing other stimuli except stimulus D. These findings suggest that the relatively high discriminability of stimulus A led to the observed differences in feedback-based learning between the two task version groups during the training phase of the PST in experiment 1. These differences in learning, in turn, may explain the observed strong relationship between stimulus-to-feedback mapping and whether or not an individual is classified as a positive or negative learner. Specifically, high discriminability of stimulus A in task version 1 in Experiments 1a and 1b led to more choices for A and therefore to a relatively high amount of positive feedback for stimulus A compared to the other training stimuli, especially compared to C. This, in turn, resulted in a positive learning bias (i.e., more accurate choices of A over other stimuli during test). In contrast, low discriminability of stimulus A combined with salience of C in task version 2 may have led to equal training performance for AB as for CD. This in turn resulted in less experienced positive feedback for A (relative to C) in version 2, and to more experienced negative feedback for B (relative to D). This, in turn, may have resulted in relatively few choices for A during the test phase as well as increased avoidance of B, and therefore in a negative learning bias (i.e., more avoidance of B compared to choosing A during test).

Moreover, results of the discrimination experiment show that stimulus character D is also relatively easy to discriminate compared to the other stimuli. This fits fairly well with the performance data of the training phase in Experiments 1a and 1b. Participants more readily selected this character when it was the more rewarding C stimulus during training in task-version 2, where it was rewarded in 70% of the trials. This explains the relatively high amount of positive feedback during the training phase for the CD pair in task version 2. In contrast, participants were less inclined to select the more rewarded stimulus C from the CD pair in version 1, presumably because it was less discriminable.

It is thus possible that the relatively weak inclination to choose A during the test phase in task version 2 reflects a relatively strong inclination to choose stimulus C in AC pairs. Thus in version 1, subjects might have learned both greater positive value for A and simultaneously, less positive value for C, facilitating their ability to choose A over C; the reverse bias in version 2 might effectively yield a more positive learned value for C than for A.

To test this idea, and to confirm the findings of the first study, we conducted a third experiment with a large sample size using a web-based implementation in samples of American rather than Dutch subjects. Along with the first two task versions, a third task version was implemented. Here, instead of swapping all of the characters (as in version 1 vs 2), we maintained the same Hiragana stimuli for the CD and EF pair as in version 1, but only swapped the A and B stimuli (i.e., to the mappings used for version 2). The logic here was that the *asymmetry* in discriminability between A and C was the main factor driving the large differences in versions 1 and 2 with opposite learning biases, and hence if these were matched such that A and C were both less discriminable, this should produce an intermediate effect with no clear bias one way or the other. Indeed, this would result in low-salient A and C stimuli that are mostly rewarded, and high-salient B and D stimuli being mostly punished. In turn this would prevent a salience-driven bias across subjects to systematically choose A or avoid B during testing.

## Method–experiment 3

### Participants

A sample of 300 participants were recruited via Amazon’s Mechanical Turk (AMT). Participants were paid $3 for their time. Based on self-report, subjects were required to be between 18–40 years of old, fluent in English, to have no history of brain injury, no medical history of mental/psychiatric disorders or drug/alcohol abuse, and were only able to complete the task once. In experiment 3 five versions of the PST were compared: version 1 and 2 with a short response window (1000 ms), version 1 and 2 with a long response window (4000 ms) and version 3. Participants completed one of these five task versions. Participants (n = 70) in version 1 long included 42% female and mean age was 31.3 (SD = 5.7). Version 1 short (n = 56) included 48% female, mean age was 31.8 (SD = 5.6). Version 2 long (n = 62) included 37% female and mean age was 30.4 (SD = 5.7). Version 2 short (n = 59) included 44% females and mean age was 29.4 (SD = 5.1). Version 3 (n = 53) included 42% female and mean age = 29.7 (SD = 5.2). Experiment 3 was approved by the Brown University institutional review board. Participant identities were protected. Participants provided their consent online by clicking ‘I Agree’ after reading the study information and consent language in accordance with procedures approved by Brown University.

### Task and procedure

Data were collected using the Amazon Mechanical Turk platform. Subjects completed a practice phase with verbal instructions with example stimuli (not used in the actual task) using deterministic feedback. Based on previous studies using AMT [34,35], participants were given a 5-item basic true-false comprehension test regarding the rules of the task. Failure to answer the questions correctly resulted in subjects repeating the practice phase and instructions.

Task versions 1 and 2 were identical to the ones described under experiment 1a and 1b. The only exception was that in the long-response-window versions (comparable to those in experiment 1b), maximal stimulus duration was not 750 ms but 4000 ms, effectively covering the complete response window, as is typical for behavioral studies with this task (although note that response times are typically < 1.5s and hence stimulus duration is rarely greater than 2000 ms). For task version 3 the CD and EF pairs were as in version 1, but the AB pair was used from version 2 (see logic for this manipulation above). A response window (and maximal stimulus duration) of 4000 ms was implemented in task version 3.

### Data reduction and analysis

Participants were excluded from the data analysis if they missed or responded faster than 200 ms to more than one-third of learning or test phase trials, and if they performed at or worse than chance in test phase training pairs. The final analysis included 58 out of 70 participants in version 1-long, 46 out of 56 in version 1-short, 46 out of 62 in version 2-long, 48 out of 59 in version 2-short, and 42 out of 53 in version 3.

Compared to the relatively extensive data analysis presented for Experiments 1a and 1b, the data from Experiment 3 were subjected to a concise and maximally surveyable test procedure addressing the essential hypotheses. To corroborate the findings of Experiment 1a and 1b we first examined the effect of stimulus mapping and presentation duration on learning bias. The overall percentages of choosing stimulus A and avoiding stimulus B in novel test pairs (A or B paired with either C, D, E or F) during the test phase were computed for each subject. Bias scores were subsequently computed by subtracting the percentage avoid-B from the percentage choose-A. These scores were entered in an ANOVA with task version (version 1 and 2) and response window (short, long) as between-subject variables.

Next, we sought to assess whether the effects of stimulus-to-feedback mapping on test phase performance as observed in Experiment 1a and 1b could be explained more proximally by differences in the reinforcement history experienced by the different groups, as suggested above. Specifically, we examined the difference in rewards experienced for A compared to the next most rewarding item C (and conversely the number of punishments experienced for B compared to the next most punishing item D). We, therefore, subtracted the percentage choose-C in CD pairs from the percentage choose-A in AB pairs in the last training block. These percentages were entered in an ANOVA with task version (version 1 and 2) and response window (short, long) as between-subject variables.

To directly test whether the critical factor in test phase bias was the relative experience of feedback for A compared to other stimuli an ANOVA was run comparing AB-CD performance for the last training block and test phase bias scores between the three task versions (now also including task version 3).

## Results–experiment 3

**Fig 6:**
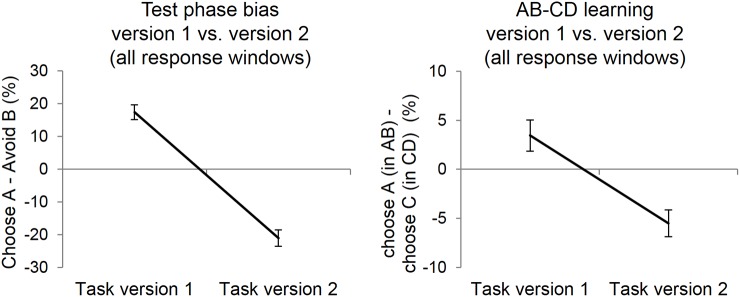
Stimulus properties affect positive and negative learning bias and choices during the training phase (experiment 3: large international sample of participants, web-based study). Left: replicating the results of experiment 1a and 1b, subjects in version 1 of the PST were better at choosing A during test phase whereas subjects in task version 2 were better at avoiding stimulus B. Right: replicating the results of experiment 1a and 1b, test phase bias was accompanied by group differences during training. In task version 1 participants performed better on the AB pair relative to the CD pair, whereas the opposite pattern was observed for task version 2. There were no significant interactions with the time to respond (1000 ms or 4000 ms). Data is therefore collapsed across both time windows. Error bars represent ± 1 SE.

First, in order to determine whether the results of Experiment 1a and 1b were replicated in a larger sample we compared test phase bias between the two task versions presented previously (version 1 vs. version 2), with two different response windows (long; up to 4000 ms vs. short; up to 1000 ms). Replicating the results of Experiment 1, we found a main effect of task version on choose A-avoid B bias (F(1,194) = 127.2; p < .001, *η*_p_^2^ = .4). As can be seen in Fig 6, left panel, percentage choose A compared to avoid B was higher for version 1 but lower for version 2. Furthermore, stimulus presentation duration had no effect on choose A-avoid B bias (F(1,194) < 0.04; p = 0.86) and did not interact with task version group (F(1, 194) = 0.1; p = 0.75), confirming that the previous results are not dependent on the specific timings used in the PST.

The ANOVA comparing AB-CD performance during training between task version 1 and 2 showed that there was a strong main effect of task version (F(1,194) = 16.5; p < .001, *η*_p_^2^ = .08). Again, participants in the version 1 group (most of them with a positive test bias, choose A > avoid B) also chose A over B more (and hence received comparatively more positive feedback) than C over D. Conversely, participants in the version 2 group (most of them with a negative test bias, choose A < avoid B) selected B over A more than D over C during training and experienced more negative feedback for doing so (Fig 6, right).

**Fig 7:**
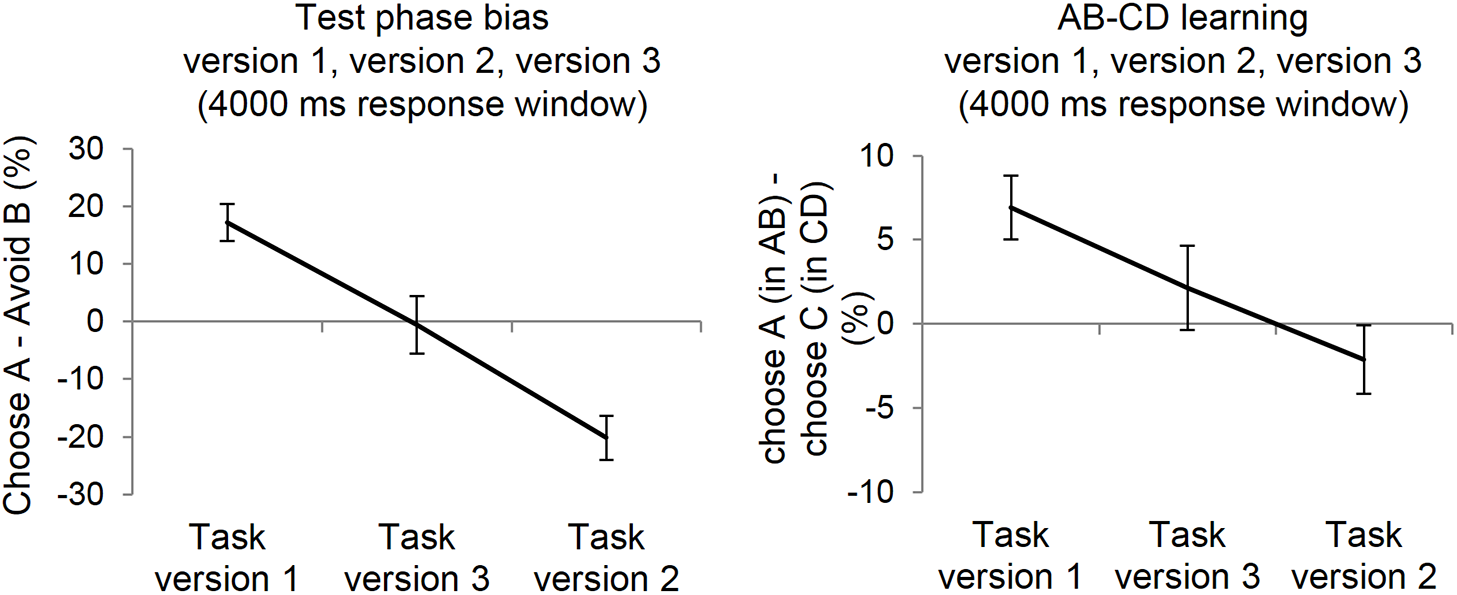
Relative experience of feedback for A compared to all other stimuli critically determined test phase bias (experiment 3). Left: participants in task version 3 in which the A and C stimulus were more comparable in discriminability showed no overall test phase bias, and it was significantly lower than in version 1 and higher than version 2. Right: there was a main effect of task version group in last learning block AB vs. CD performance. Participants in task version 3 performed equally well on the AB and CD pair in contrast to participants in task version 1 and 2. The task versions presented had a response window of 4000 ms. Error bars represent ± 1 SE.

For the comparisons between the three groups we only used the long (4000 ms) response window/ presentation duration as the previous analysis showed that presentation did not impact test-phase bias and hence version 3 was only run with this standard presentation. Results from an ANOVA showed a main effect of task version on AB vs. CD choices during the last learning block (F(2,143) = 4.78; p < 0.01, *η*_p_^2^ = .06; Fig 7, right) and test-phase bias (F(2,143) = 23.62; p < .001, *η*_p_^2^ = .25; Fig 7, left panel). The effect of task version on AB-CD choices during training was driven by a significant difference between version 1 and 2 (p = .002). There was no significant difference between task version 3 and the other versions (v1 vs 3: p = .13; v2 vs 3: p = .19).

Critically, group 3 showed no overall test- phase bias, and this bias was significantly lower than that for group 1 (v1 vs. v3: t(98) = 3.11; p = .002, d = .63) and higher than that for group 2 (v3 vs. v2: t(86) = 3.14; p = .002, d = .67). This ranking of version1>version3>version2 in the test phase bias confirms our hypothesis that the critical factor driving test phase biases is relative discriminability and hence reinforcement history for A/B compared to C/D.

## Discussion–experiment 3

The results of Experiment 3 replicate the findings of Experiment 1a and 1b in a large scale web-based sample and provide further evidence that test-phase bias is strongly predicted by stimulus-to-feedback-mapping. These experiments also show similar between-group differences in learning curves for each of the training pairs, indicating that the positive/negative bias is likely explained by differences in feedback learning or experience during the training phase.

In an additional task version 3, we examined performance in a case in which only the stimuli of the AB pair were switched (i.e., CD and EF pair as in version 1, AB as in version 2). The aim was to investigate whether the absolute amount of reinforcement for stimulus A and B or the relative amount of reinforcement for A and B compared to all other stimuli was the critical factor determining positive/negative learning bias. The results indicate that the latter was the case. No clear positive or negative learning bias was observed for this particular task version, suggesting that when discriminability is matched, individual differences are more likely related to veridical differences in positive and negative learning per se.

Training performance for version 3 was in between that for version 1 and version 2 (as assessed in Experiment 3; Fig 7 right panel). Specifically, there was a large difference between AB and CD performance in version 1; a slightly reversed difference in version 2 (which means that C was chosen over D slightly more than A over B); and an intermediate (slightly positive) difference in version 3. For version 3 this resulted in an amount of experienced feedback for A (relative to C), as well as an amount of experienced negative feedback for B (relative to D) that was intermediate between that for version 1 and version 2.

This was associated in the test phase with an about equal amount of choices to avoid B and to avoid D in BD pairs. By the same token, there was an equal amount of choices for A as there were for C in AC pairs. Together this effectively annihilated the contribution of relative salience to negative or positive-learner classification.

## General discussion

The aim of the current study was to investigate if the extent to which individuals learn better from positive or negative outcomes is influenced by the extent to which aspects of the stimulus configuration afford learning from the stimuli in general. In two experiments (Experiment 1a and 1b), participants performed one of two versions of a common probabilistic selection task described by Frank et al. [5,14]. These task versions were identical except instead of randomizing or counterbalancing the stimulus assignments as is typically done, here we explicitly fixed them in each version but manipulated them across groups to study their impact. Specifically, the assignment of positive and negative feedback to the stimuli was switched, e.g., the A stimulus (rewarded 80%) in the first version became the B stimulus (punished 80%) in the second version, and vice versa. Critically, we found that during the test phase subjects almost exclusively chose A (rather than avoid B) in version 1 but avoided B (rather than choose A) in version 2. Similar results were obtained regardless of presentation duration and response windows across all experiments. When task-version-1 stimulus A was associated with mostly positive feedback, all except one (experiment 1a) or two (experiment 1b) participants were characterized as positive learners, whereas when this same stimulus became B in task version 2 and was associated with negative feedback, all except one participant (experiment 1a) or all participants (experiment 1b) were categorized as negative learners. Furthermore, these results were replicated in a large-scale web-based experiment (experiment 3).

Notably, a follow-up discrimination experiment revealed that two of the six Hiragana characters (A and D in version 1; B and C in version 2) were more discriminable than the others, as assessed by response times. Together these findings indicate that the relative perceptual discriminability of characters assigned to high or low reward probabilities strongly influenced performance.

Importantly, biased performance during the test phase of the PST was preceded by group differences in learning curves during training. These differences were consistent across all experiments. In task version 1, participants most readily selected the highly salient A over B and hence received a high amount of positive feedback for the A stimulus relative to the other training characters. This pattern was most pronounced for the task with a response window of 4000 ms. In contrast, in task version 2, participants showed comparable accuracy in AB and CD pairs, despite the lower reward probability of the latter. This implies that, relative to version 1, the amount of experienced positive reinforcement for A relative to C was reduced, and the amount of experienced negative reinforcement for B relative to D was increased. This may have resulted in a relative prevalence of C over A choices during the test phase, as well as a stronger avoidance of B over D, which in turn resulted in the inclination to more avoid B than to choose A. The latter pattern was observed for both response windows. The pattern of differences during training performance was also visible already in the start training blocks in experiments 1a and 1b, indicating that it emerged rapidly within the very first ten repetitions of each stimulus and associated feedback.

Experiment 3 was conducted in order to test whether these findings were robust by replicating the experiment in a larger sample. Indeed, experiment 3 confirmed the interpretation in terms of relative discriminability and feedback. Moreover, task version 3 was developed to more specifically engineer a stimulus mapping that would produce less bias under this interpretation. In this version, relative to version 1, only the stimuli within the A-B pair were switched, thus better matching the discriminability of A vs C, and indeed no learning biases were observed in the test phase. The results indicate that the relative amount of reinforcement for A and B compared to all other training stimuli influenced learning bias. Specifically, in version 3 the relative AB–CD performance difference during learning–and hence the degree to which A was reinforced more than C and B punished more than D–was intermediate between version 1 and version 2. In the test phase this resulted in an about equal amount of choices to avoid B and to avoid D, and to an equal amount of choices for A as there were for C.

Together, our findings indicate that stimulus salience/discriminability strongly affects preference behavior during reinforcement learning. A highly salient stimulus that is most frequently rewarded rapidly attains a preferred status (perhaps due to being more memorable), is chosen much more often, and as such obtains a higher learned value, than a less salient stimulus that is less frequently rewarded. In contrast, a relatively less salient stimulus that is most frequently rewarded attains a level of preference that is comparable to or even lower than that of a highly salient stimulus that is less frequently rewarded.

On a more general level our results are consistent with prior studies indicating that stimulus salience and value information interact to bias learning and decision making [28–32]. Our analysis of discriminability in relation to learning also fits earlier accounts that stress the importance of similarity among less discriminable objects rather than the distinctiveness of more discriminable objects (e.g.,[36]). As argued in the discussion of Experiment 2, the four less discriminable stimuli in our setup can be considered mutually less discriminable and therefore relatively similar or homogenous, compared to the two stimuli that were more discriminable relative to the less discriminable ones. To the extent that superior learning for salient items actually depends on the homogeneity of the non-salient items (as, e.g., in the von Restorff effect), this homogeneity was actually realized in the present training sessions.

On a more operational level our results imply that one needs to exert caution in interpreting data from individual subjects in the PST and comparable reinforcement learning tasks (e.g., [4,5,7,8]). Note that the cited studies using the PST counterbalanced or randomized the stimulus mappings across subjects, and hence previous replicated findings of individual differences due to genetics [10,12,13,17], striatal D1 and D2 dopamine receptor binding [11], and neural responses to feedback [9,14,17,19] cannot be attributed to discriminability, given that these observed effects held despite stimulus counterbalancing, and largely were present without differences in learning curves (in contrast to effects of discriminability seen here). However, the present results suggest that first, such counterbalancing is paramount; second, that test phase performance differences should always be accompanied by analysis of learning curves during training; and third, that it is difficult to interpret the results of any one individual (as opposed to a group of individuals with e.g. same genotype) unless care is taken to match the discriminability of the stimuli.

On the other hand, the results of the third version in effect point the way to the implementation of the PST in a manner that reduces the contribution of stimulus salience to classification as either negative or positive learner, at least at the level of group averages, by matching discriminability. Indeed an initial analysis of our other online datasets using everyday geometric shapes that are all highly discriminable (e.g. blue square, red triangle etc.), revealed no effect of stimulus assignment on learning bias (unpublished data). The very possibility of such a contribution, as well as the solution proposed here, have ramifications for any task variety in which per subject a fixed relation between physically different stimuli on the one hand, and feedback conditions on the other is maintained (see, e.g., the references to other PST varieties provided in the Introduction).

It may also be noted that some tasks use the very same stimuli that are assigned to both reward and punishment, where those values alternate across blocks (as in reversal learning). In this case there are no differences in discriminability across conditions. Notably, much like the PST, individual differences in sensitivity to positive and negative outcomes in reversal tasks are related to individual difference in striatal dopamine levels assessed with PET and these biases are similarly affected by dopaminergic medications [37,38].

Nevertheless, the current data suggest that differences in the discriminability of stimuli can add significant noise to the measurement of individual differences in learning style. In previous studies using the PST or a comparable reinforcement-learning task, participants likely also integrated sensory evidence and value to form their own estimates of expected reward (or punishment). It is notable in this respect that a recent ERP study found that the ERN is modulated by both stimulus salience and reward level [31], in line with the notion that both the perceptual properties of stimuli and their value influence probabilistic learning and future decision making.

Although there was a strong overall relationship between salience-reward contingencies and outcome as positive/negative learner, not all participants subjected to version 1 of the reinforcement task (in which a highly salient stimulus was associated with positive feedback) were classified as positive learner in our study. In the same vein, not all participants subjected to version 2 (in which a highly salient stimulus was associated with negative feedback) were classified as negative learner. Thus, while our results indicate that differences in stimulus discriminability bias the outcome of the reinforcement learning task in terms of ‘positive’ or ‘negative’ learning style, stimulus discriminability is not the only factor that determines learning style. This is again in line with recent work suggesting that stimulus salience and stimulus value both affect learning about expected reward (or punishment) [28–32].

In sum, the current study shows that differences in stimulus salience /discriminability are a potential confounding factor in reinforcement learning tasks during both learning and transfer phases. The extent of selecting rewarding stimuli or avoiding punished ones depends on the relative salience of these stimuli, both during and after learning. To avoid these potential confounds in future research, task stimuli should be matched at the individual level in terms of relative discriminability; learning curves should always be assessed before interpreting test phase data, and/or a larger number of stimuli should be used within subject having similar values. On a more theoretical note, the present study elucidates how reinforcement and punishment learning are influenced by the relative salience tied to choice alternatives. This extends a growing body of both older (e.g., [23,24]) and recent literature on the relation between salience and learning.

## Supporting information

S1 File Alternative analysis experiment 2. (DOCX)
