# Supplementary material for: Stimulus discriminability may bias value-based probabilistic learning
Source: PLoS One. 2017 May 8;12(5):e0176205. doi: 10.1371/journal.pone.0176205 (PMC5421749; doi:10.1371/journal.pone.0176205)
Supplement: S1 File — (DOCX) [file pone.0176205.s001.docx]

Alternative analysis Experiment 2

The data were also analyzed in a different manner, namely by only including trials with the presentation of a specific stimulus and by subsequently averaging these data across all conditions with that specific stimulus. Differences as revealed by this analysis may reflect not only discriminability but also differences in preference. This may hold to the extent that certain single specific stimuli are responded to more quickly not only because they are more discriminable, but also because of their inherent likeability, inducing a more vehement approach tendency. Six average RTs for each subject (one for each Hiragana character) were entered into a MANOVA. The results of this analysis yielded very similar results compared to the analysis reported in the Results section of Experiment 2, which indicates that preference or approach does not have an additional impact on reaction times and that discriminability is the exclusive driving factor for the observed differences.
